# Bilateral breast infection following total breast reconstruction with autologous fat transfer (AFT): A case report

**DOI:** 10.1016/j.ijscr.2023.107917

**Published:** 2023-02-03

**Authors:** Alexander Gabriël Saelmans, Maud Rijkx, Juliette Hommes, René van der Hulst, Andrzej Piatkowski

**Affiliations:** Department of Plastic, Reconstructive and Hand Surgery, Maastricht University Medical Centre, 6229 HX Maastricht, the Netherlands

**Keywords:** Breast infection, Pre-expansion device, Fat transfer, Lipofilling, Breast reconstruction, Case report

## Abstract

**Introduction and importance:**

Total breast reconstruction with autologous fat transfer (AFT) has a low complication rate. Fat necrosis, infection, skin necrosis and hematoma are the most common complications. Infections are usually mild and manifested by a unilateral red painful breast and treated with oral antibiotics with or without superficial irrigation of the wound.

**Case presentation:**

One of our patients reported an ill-fitting pre-expansion device several days after surgery. This was due to a severe bilateral breast infection following a session of total breast reconstruction with AFT despite perioperative and postoperative antibiotic prophylaxis. Surgical evacuation was performed in combination with both systemic and oral antibiotic treatment.

**Clinical discussion:**

Most infections can be prevented in the early post-operative period with antibiotic prophylaxis. If an infection does occur, it is treated with antibiotics or superficial irrigation of the wound. A delay in identification of an alarming course could be reduced by monitoring the fit to the EVEBRA device, implementing video consultations on indication, limiting the means of communication and better informing the patient on what complications to monitor. The recognition of an alarming course following a subsequent session of AFT is not guaranteed after a session without complication.

**Conclusion:**

Besides temperature and redness of the breast, a pre-expansion device that doesn't fit can be an alarming sign. Patient communication should be adapted as severe infections can be insufficiently recognized by phone. Evacuation should be considered when an infection does occur.

## Highlights

• Informing patients on what complications to monitor should be kept concise and understandable to a layperson.
• Video consultations on indication could reduce a delay in identification.
• The recognition of an alarming course is not guaranteed following a complication-free session.
• An ill-fitting pre-expansion device can be an alarming sign.
• Evacuation should be considered when an infection does occur.

## 1 Introduction

In autologous fat transfer (AFT) fat tissue is harvested from donor sites, processed and reinjected into the recipient site. Lipofilling is used as an adjuvant procedure to refine other breast reconstruction techniques as well as a standalone total autologous breast reconstruction technique [1,2]. Total breast reconstruction with AFT has proven to be satisfying and safe [3,4]. This technique has multiple advantages including the minimal invasiveness of the procedure, fat harvesting through liposuction from unappealing parts of the body, relatively short operation time and brief hospital stay. However, the need for a series of operative procedures and the limited maximum achievable breast size are disadvantages of the technique [3,[5], [6], [7]].

Autologous total breast reconstruction with AFT has a low complication rate. Fat necrosis and infections are the most common complications following AFT. Both complications will clinically present as a lump after breast surgery. Besides the presence of a lump, an infection presents as indurated and painful. These infections are mostly treated with oral antibiotics and are usually simple to manage and benign. Other possible complications are hematoma and skin necrosis. Significantly more complications after AFT are observed in patients who smoked or underwent chemo- or radiation therapy [5,6,8,9].

Bilateral breast infection after a session of total breast reconstruction with AFT has seldomly been reported in literature. Herein, a case of bilateral breast infection following autologous fat transfer to the breasts is described. This case report has been reported in line with the SCARE Criteria [10].

## 2 Presentation of case

A 46-year-old non-smoking female carrier of the BRCA1 mutation with a body mass index of 26 kg/m^2^ underwent a prophylactic bilateral nipple-sparing mastectomy in October 2019. Following the mastectomy, the breast skin was expanded with tissue expanders during six weeks on the right side and eight weeks on the left side in another hospital. The right tissue expander was removed, because of impending skin necrosis. The left tissue expander was removed to eliminate asymmetry. Two years after bilateral removal of the tissue expanders, the patient was included in the BREAST II-EVE study, a multicentre prospective cohort study for total breast reconstruction with AFT in the Netherlands. In addition, the EVE-study is a randomized study within the BREAST II study that aims to assess the effect of an external vacuum expander (EVE) on this breast reconstruction method (ClinicalTrials.gov Identifiers NCT02339779, NCT04261829).

A second session of bilateral AFT grafting was performed three months after her first uncomplicated session. Perioperatively, intravenous antibiotic prophylaxis with 2 g of Cefazoline was administered. Before initiating the surgery, disinfection was carried out with iodine alcohol solution. The operation was performed by a plastic surgeon with 10 years of experience with total breast reconstruction with AFT in a university hospital. Saline, adrenaline solution was used bilaterally for infiltration of the donor sites, the trochanter and femur regions bilaterally. Using the Lipografter® (MTF Biologics, Edison, United States), 800 ml of fat (and saline solution) was harvested bilaterally. Consecutively, the left breast was filled with 340 ml and the right breast with 380 ml of purified fat. Concurrently, rigotomy of the scar and inferior pole took place following lipofilling. The donor site incisions were closed with a resorbable 5-0 monofilament thread. The recipient site incisions were covered with Terra-Cortril, an antibiotic ointment, once post-operatively and a sterile gauze. Orally administered antibiotic prophylaxis with amoxicillin/clavulanic acid 500 mg/125 mg three times daily was prescribed for five days. From the fifth postoperative day onwards, the patient had to wear the EVEBRA device for two weeks with the curvature at the height of the inferior pole.

A standard follow-up visit in the outpatient clinic is scheduled about two weeks after the surgery. If problems occur, patients are advised to contact the clinic beforehand by phone. The patient informed the department of plastic surgery on postoperative day 5 by email that she was not going to wear the EVEBRA device, because the reconstructed breast didn't fit to the EVEBRA device anymore. No other symptoms were mentioned. On postoperative day 7, the patient reached out to the outpatient clinic by phone. A small wound dehiscence of the upper side of the right breast with effusion of wound fluid was observed by the patient eight days after surgery.

The patient requested to be seen on postoperative day ten by a plastic surgeon because of worsening of the wound dehiscence and effusion. During the consultation, the patient presented with headache, nausea, shivering and a loss of appetite. Physical examination revealed preference for a stooped position, tachycardia (114 bpm) and fever (38.6 °C). Other vitals were normal. Additionally, inspection of the right breast showed suppurating wound fluid with a pungent smell and was suggestive for hematoma and fat necrosis. The left breast tissue fluctuated and was tense, but needle puncture didn't result in depressurization. Wound fluid and blood-samples were taken for additional analysis. The value of haemoglobin was 6.1 mmol/l.

The 46-year-old patient was admitted to the plastic surgery ward and underwent a bilateral surgical evacuation of hematoma and fat necrosis. A breast infection caused by infected hematoma in combination with fat necrosis was perceived as most probable indication for treatment. Perioperatively, administration of intravenous antibiotic prophylaxis with 2 g of Cefazoline took place. On the right breast, signs of active bleeding required a larger incision of the breast and haemostasis. The left breast was leaking deliquescent fat necrosis with a pungent smell out of a small incision made lateral to the areola without any signs of active bleeding. Then, the breasts were flushed with a sodium chloride solution and suction drains were placed before closing the breasts with non-resorbable 4-0 thread mattress sutures. A pressure band was worn postoperatively.

**Fig. 1:**
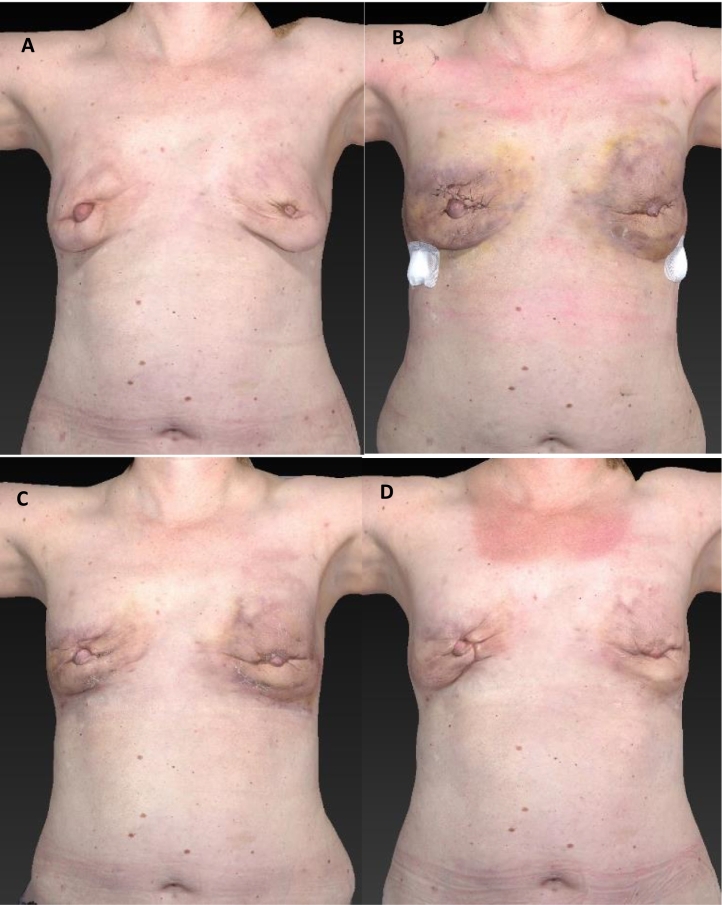
**A** Nine weeks following the first session of AFT (three weeks previous to the second session of AFT) **B** Four days following the evacuation (fourteen days following the second session of AFT), drain incisions are covered with band aids **C** Fourteen days following the evacuation **D** Two months following the evacuation.

In line with expectations, extensive ecchymosis and diffusely oedematous hardened breasts were observed in the postoperative days (Fig. 1). A positive wound culture revealed predominantly *Enterobacter cloacae* complex, resistant to amoxicillin/clavulanic acid, and a limited amount of *Staphylococcus intermedius*. No pathogens were detected in the blood culture. Amoxicillin/clavulanic acid was administered intravenously for three days. In the six days afterwards, the administration switched to oral antibiotic therapy. The two drains produced serosanguineous fluid and were removed on the fourth day after the operation. On day four after surgical evacuation the patient was discharged to home.

One month after the infected hematoma evacuation, follow up ultrasonography showed small areas of fat necrosis and a bilateral basis of fat. No advancement in volume, using VECTRA 3D imaging, was seen after six weeks (Table 1). A third session of AFT was postponed to at least 3–4 months after the evacuation surgery.

**Table 1:** Breast volumes (cm^3^) of the patient measured with VECTRA 3D imaging at various stages of total breast reconstruction with AFT.

Breast reconstruction stage	Right breast volume (cm^3^)	Left breast volume (cm^3^)
*First session of AFT*		
Preoperatively without expansion	64.8	57.7
9 weeks postoperatively	255.9	153.0

*Second session of AFT*		
9 weeks postoperatively	138.5	144.7

## 3 Discussion

Until recently, lipofilling was used as an add-on to decrease remaining smaller deformities in an area of the breast. There is a substantial increase in the number of total breast reconstructions with AFT exclusively. In AFT, fat tissue is harvested by means of liposuction from donor sites. Subsequently, liposuctioned fat tissue is processed and reinjected into the recipient site, the breast subcutaneous tissue, for total breast reconstruction. Multiple sessions are required to obtain the desired result. To pre-expand the breast before a session of AFT, an EVE-cup can be used. Survival of the injected fat cells is facilitated by angiogenesis [6,11].

The relatively low infection rate of total breast reconstruction with AFT is reflected by the occurrence of only six local unilateral infections at the recipient site, treated with oral antibiotics, in a study that included 880 fat graft procedures [12]. In the literature, surgical treatment in severe infection after a session of AFT has been described in four case reports [[13], [14], [15], [16]]. Remarkably, these women had bilateral mammary abscesses. In septic patients, needle aspiration drainage, incision and drainage, intravenous antibiotics and daily irrigation brought the infection under control [[13], [14], [15]]. In a less urgent case with increased aesthetic interest, pus can be removed by ultransonography (US)-guided aspiration [16]. In general, infections following AFT mostly occur following aesthetic surgery as reported several times in literature. Many are associated with atypical (Non-Tuberculous) Mycobacteria. Cosmetic surgery is frequently sought in the countries with endemic distribution [17]. It is unclear what caused a bilateral breast infection in the aforementioned 46-year-old patient. Contamination from incomplete sterilization or cellulitis are possible causes. Also, an occult seroma could have facilitated the infection after the surgery.

Administration of oral antibiotic prophylaxis in patients is mostly applied following total breast reconstruction with AFT. An anti-apoptotic effect exerted by certain antibiotic elements is hypothesized, maximizing the safety of the procedure. Currently, no guidelines are available concerning the use of prophylactic antibiotics in AFT. A retrospective multicentre study by Morandi et al. revealed that complication rate in breast reconstruction with AFT is not lowered by prolonging prophylactic antibiotic administration. Also, the complication rate is not correlated to the antibiotic administration period. Based on this study, a single shot perioperative antibiotic seems to be an alternative to postoperative antibiotics. Additionally, most patients received the antibiotic prophylaxis for 5–6 days following surgery. Second-generation cephalosporins, predominantly cefuroxime, are most prescribed according to this study [9].

The EVEBRA device was not worn by the patient because of an inadequate fit and lack of comfort. This should have been alarming in this early reconstruction phase. External pre-expansion systems should be tailored specifically to facilitate the volumetric requirements needed to achieve reconstruction for each patient. The use of these devices is limited to clinical trials in Europe. Complications can also be caused by the system itself in as many as 25 % of patients [18]. Dermatitis was reported in as many as 79 % of treated cases, and cellulitis in 14.3 % in a case series of 11 patients [19]. There was a clear delay between the first signals, reported by the patient, and the identification, by the medical staff, of an alarming course. When it comes to follow-up consultations, there is a trade-off between a higher follow-up frequency, possibly resulting in earlier detection, and the desire to reduce the visits as much as possible in the context of time efficiency. Telemedicine on indication, e.g. a video consultation, could be a compromise [20]. A video consultation would provide additional information, while preventing unnecessarily seeing patients in the outpatient clinic, thus saving time. Furthermore, for patients it might be confusing what means of communication, phone or email, to use to report problems. This confusion could be removed by limiting the means of communication to one. Also, informing patients on what complications to monitor should be kept concise and understandable to a layperson.

Reflecting on the complicated patient journey, we are surprised that the patient noticed a difference between the recovery after the first and the second session of AFT, but did not act seriously until day 10. The skin was shiny and dehiscence took place. However, this was not reported to the assistant before the admission to the hospital. The patient thought it was within the postoperative normal range, although she was seriously ill.

## 4 Conclusion

Since rapid intervention is important for graft survival, providing information concerning what complications to monitor, limiting means of communication, video consultations on indication and monitoring the fit to a pre-expansion device could prove to be useful in earlier detection of infections. Having undergone a complication-free session of AFT, does not guarantee the recognition of an alarming course following a subsequent session by the patient. When an infection does occur while taking antibiotics, evacuation should be considered.

## Consent

Written informed consent was obtained from the patient for publication of this case report. A copy of the written consent is available for review by the Editor-in-Chief of this journal on request.

## Ethical approval

The study is exempt from ethical approval in our institution.

## Funding

Not applicable.

## Guarantor

Andrzej Piatkowski.

## CRediT authorship contribution statement

Alexander Gabriël Saelmans, B.S.: study concept, design and write the paper
Maud Rijkx, M.D.: study concept and write the paper
Juliëtte Hommes, Ph.D., M.D.: study concept and write the paper
Andrzej Piatkowski de Grzymala, Ph.D., M.D.: study concept and write the paper
René van der Hulst, Ph.D., M.D.: write the paper.

## Conflicts of interest

Not applicable.
